# Correlation of dystonia severity and iron accumulation in Rett syndrome

**DOI:** 10.1038/s41598-020-80723-1

**Published:** 2021-01-12

**Authors:** Tz-Yun Jan, Lee-Chin Wong, Ming-Tao Yang, Chien-Feng Judith Huang, Chia-Jui Hsu, Steven Shinn-Forng Peng, Wen-Yih Isaac Tseng, Wang-Tso Lee

**Affiliations:** 1 Graduate Institute of Brain and Mind Sciences, College of Medicine, National Taiwan University, Taipei, Taiwan; 2 Department of Pediatrics, Cathay General Hospital, Taipei, Taiwan; 3 Department of Pediatrics, Far Eastern Memorial Hospital, New Taipei City, Taiwan; 4 Department of Chemical Engineering and Materials Science, Yuan Ze University, Taoyuan, Taiwan; 5 Institute of Medical Device and Imaging, National Taiwan University College of Medicine, Taipei, Taiwan; 6 Institute of Biomedical Engineering, National Taiwan University College of Medicine, Taipei, Taiwan; 7 Department of Pediatrics, National Taiwan University Hospital SinChu Branch, Taipei, Taiwan; 8 Department of Medical Imaging, National Taiwan University Hospital and National Taiwan University College of Medicine, Taipei, Taiwan; 9 Molecular Imaging Center, National Taiwan University, Taipei, Taiwan; 10 Department of Pediatrics, National Taiwan University Hospital and National Taiwan University College of Medicine, 8, Chung-Shan South Road, Taipei, 100 Taiwan

**Keywords:** Neurology, Signs and symptoms

## Abstract

Individuals with Rett syndrome (RTT) commonly demonstrate Parkinsonian features and dystonia at teen age; however, the pathological reason remains unclear. Abnormal iron accumulation in deep gray matter were reported in some Parkinsonian-related disorders. In this study, we investigated the iron accumulation in deep gray matter of RTT and its correlation with dystonia severity. We recruited 18 RTT-diagnosed participants with *MECP2* mutations, from age 4 to 28, and 28 age-gender matched controls and investigated the iron accumulation by susceptibility weighted image (SWI) in substantia nigra (SN), globus pallidus (GP), putamen, caudate nucleus, and thalamus. Pearson’s correlation was applied for the relation between iron accumulation and dystonia severity. In RTT, the severity of dystonia scales showed significant increase in subjects older than 10 years, and the contrast ratios of SWI also showed significant differences in putamen, caudate nucleus and the average values of SN, putamen, and GP between RTT and controls. The age demonstrated moderate to high negative correlations with contrast ratios. The dystonia scales were correlated with the average contrast ratio of SN, putamen and GP, indicating iron accumulation in dopaminergic system and related grey matter. As the first SWI study for RTT individuals, we found increased iron deposition in dopaminergic system and related grey matter, which may partly explain the gradually increased dystonia.

## Introduction

Rett syndrome (RTT), a rare neurodevelopmental disease, is typically delineated by developmental variation in cognition, hand use and motor functions. This disease involves approximately 1:9–10,000 female births^1–3^, most of whom demonstrate a mutation in the MECP2 (methyl-CpG-binding protein 2) gene which discovered by Amir et al.^1,4–7^. Although individuals with RTT exhibit relatively normal infant development^8–11^, they manifest stereotypical hand movements and gait abnormalities with poor balance between ages 1 and 4 years^12^. Between ages 2 and 10 years, symptoms stabilize after the period of regression. Although some individuals with RTT may remain at this stage for the rest of their lives, others may deteriorate from the teen years or early twenties, showing muscle weakness, rigidity, and spasticity and developing bradykinesia, scoliosis, and distal-limb distortion due to a long period extrapyramidal involvement^13,14^. Parkinsonian features may also develop with dystonia in the limbs and the head and neck. These symptoms are possibly correlated with nigrostriatal–dopaminergic pathway dysfunction and abnormal MECP2 input^15,16^. The possibility of premature aging was also suggested^17^.

However, the exact cause of degeneration and parkinsonian features in RTT is not clear. Abnormal iron accumulation in deep brain nuclei was found in a heterogeneous group of neurodegenerative diseases with brain iron accumulation (NBIA), characterized by extrapyramidal movements. Individuals with NBIA may show approximately three times the normal deposition of iron in the globus pallidus (GP) and substantia nigra (SN) but normal amounts in other brain regions^18,19^, indicating that accumulation of iron in deep brain nuclei may associate to extrapyramidal symptoms. Iron may also accumulate in brain structure of healthy people due to aging. A postmortem study revealed that iron was accumulated at a higher level in extrapyramidal structures than in the cortex^20^. Susceptibility weighted imaging (SWI) studies also revealed that mineralization of the deep gray matter increased with age from infancy to late adulthood^21–23^. Excessive iron deposits may induce oxidative stress and damage mitochondrial function in neurons. Therefore, the over-accumulation of iron in deep brain nuclei may cause irreversible cellular injury, leading to dystonia or parkinsonian features.

Recent studies investigating differences in iron deposition in Parkinson’s disease (PD) and atypical PD found increased iron in the SN and caudate nucleus (CN) in PD and in the putamen and GP in atypical PD. Iron content was also correlated with clinical severity^24,25^. Recently, the effect of extraordinary iron accumulation in other neurological diseases mimicking atypical RTT has been deliberated. An individual with WDR45 mutation was reported to have signal loss in SN and GP from T1 and T2* weighted imaging^26^. However, age-related changes in iron content in RTT and its correlation with parkinsonian features and dystonia severity in typical RTT remain unclear. Therefore, the primary purpose of this study was to depict iron deposition in the deep gray matter in RTT and investigate its relation with dystonia severity. We used a ferromagnetic-sensitive technique, SWI, to gain information about mineralization in the basal ganglia in individuals with RTT. We hypothesized that iron accumulation in the deep gray matter may be related to dystonia severity in patients with RTT.

## Method

### Participants

A total of 18 female RTT patients with MECP2 mutations were recruited. All study participants were enrolled from the Joint Clinic for Rett syndrome in National Taiwan University Hospital and were diagnosed to be RTT based on the revised diagnostic criteria^27^ by the major neurologist (Lee W.T.). We further subdivided the patients with RTT into two age groups (age < 10 years and age > 10 years). Twenty-eight age- and gender-matched healthy controls without neurologic diseases (e.g., psychiatric diagnoses, history of neurological impairment, neuropsychiatric conditions, or genetic disorders) were also enrolled for comparison. Patients with RTT were sedated with chloral hydrate before scanning to avoid motion artifacts. During the scanning period, heart rate and pulse oximetry were continuously monitored. The study was approved by the Institutional Review Board of the National Taiwan University Hospital, and informed consent was obtained from all participants or their legal guardians (201510011RINC). All methods were performed in accordance with the relevant guidelines and regulations.

### MRI protocol

In the present study, we utilized SWI to acquire information on abnormal metal concentrations in the deep gray matter. The SWI method applies the principal of susceptibility difference, and tissues can be distinguished by their contrast signals in sufficiently long echo times. Iron has paramagnetic properties and increases local magnetic susceptibility when an external magnetic field is applied. Tissues containing iron will strengthen and interfere with the local magnetic field. This characteristic can be applied to quantitatively determine the iron concentration in a tissue^28,29^. To generate images, SWI uses a three-dimensional gradient echo sequence with a long echo time and flow compensation to acquire high-resolution magnitude imaging and phase imaging. Phase imaging can reflect static magnetic field inhomogeneities and be utilized as a high-pass filter to mask the original magnitude imaging by multiplication^30^.

All of the scans were acquired using a Siemens 3 T scanner (Tim Trio, Germany) with a 32-channel phased array coil. The MR imaging protocol consisted of sagittal T1-weighted, axial fast spin-echo T2-weighted imaging, and SWI with flow compensation, and the following sequences were acquired: T1 MPRAGE (slice thickness = 1.0 mm, TR/TE = 2000/2.98 ms, flip angle = 9°, FOV read = 256 × 192 mm^2^, and 208 slices per slab), T2 (slice thickness = 2.5 mm, TR/TE = 9650/103 ms, FOV = 200 × 200 mm^2^, and 56 contiguous axial-weighted images), and SWI (slice thickness = 2.5 mm, TR/TE = 28/20 ms, flip angle 15°, FOV = 200 × 200 mm^2^, and 56 contiguous axial-weighted images) with flow compensation (TR/TE = 28/20 ms, FOV = 230 × 230 mm^2^, voxel size = 0.8 × 0.7 × 1.6 mm, flip angle 15°, and 72 slices per slab with 22.2% oversampling). Images were read on a commercially available workstation of Siemens by the neuroradiologist (Peng, S. F.) for excluding the presence of infarcts and hemorrhages.

### Image analysis

Analysis of signal intensity on SWI was documented according to the anatomical structures of bilateral regions of interest (ROIs) (SN, CN, putamen, GP, and thalamus) on the slice, where they were visualized best. Visual grading scale, a semi-quantitative evaluation was applied to score the signal intensity in 10 ROIs. Hypointensity was measured according to a relative grading scale (compared with the cerebrospinal fluid, CSF) by visual discrimination, ranging from 0 to 3 points (0: SI similar to CSF intensity; 1: mild; 2: moderate; 3: severe)^31^. Additionally, to compare the inhomogeneity, a quantitative evaluation, contrast ratio was used to examine the image variation of 10 ROIs. Each ROI was measured bilaterally as signal intensity (SROI) and calculated against the occipital white matter (SO) as a contrast ratio “CR = (SROI − SO)/SO”. The occipital white matter of individuals with RTT is considered less variable, so it was chosen as a comparable signal baseline. To investigate the effect of changes in the dopaminergic system on dystonia severity, the average value of all regions in the dopaminergic system and related grey matter, including the SN, putamen and GP, was obtained as a new variable. All images were analyzed independently by an experienced neuroradiologist (Peng, S. F.), who was blind to clinical symptoms, following defined radiological protocols.

### Behavioral measurements

Parents/caregivers were requested to complete a questionnaire to collect clinical information, including age, gene mutations, motor function, and clinical features. The Rett Syndrome Gross Motor Scale includes 15 gross motor items with levels from ability to sit to challenging mobility^32^. We grouped items into four subgroups to present a general view of motor function. The hand function scale is utilized to evaluate the level of hand–eye coordination. Considering the hand function of patients with RTT, the scale was designed ranging from level 1 (“no observed hand function”) to level 8 (can orientate and determine with reasonable accuracy the position and size of objects when the hand is approaching them)^32^. For evaluation of clinical features, the Rett Syndrome Severity Scale and Rett Syndrome Behaviour Questionnaire were used for research assessment. The descriptions include the severity level of symptoms, pathological behaviors and diverse clinical features in RTT^33–35^. Lastly, severity scales of dystonia were measured by experienced neurologists. We used the Fahn–Marsden scale to evaluate dystonia in 9 body areas with a weighting factor (eyes, mouth, speech and swallowing, neck, bilateral arms, bilateral legs, trunk), which halves the contribution of dystonia in the eyes, mouth and neck^36^. Another scale, the Unified Dystonia Rating Scale, was also used to perform a more detailed assessment of body areas (e.g., to separate the item ‘face’ into ‘eye and upper face’ and ‘lower face’) and to add severity and duration ratings for a more comprehensive observation^37^.

### Statistical analysis

Clinical features of the two age groups (age < 10 years and age > 10 years) were compared by using a nonparametric Mann–Whitney U test. The nonparametric U test was also applied to assess between-group differences in signal intensity on the visual grading scale. An independent *t* test was used to compare contrast ratios between the RTT and control groups in 5 ROIs and the average value of 3 ROIs. Additionally, a multiple linear regression model was used to explore the abnormal association between signal intensity and age in the RTT group. We assigned group as a dummy variable (control = 0, RTT = 1) and used centering methodology to reduce multicollinearity with the age variable and interaction term (age × group). A full regression model containing both the main effects of age and group, as well as interaction effects, was represented by the following formula:

$${\text{Y}}_{{\text{contrast ratio}}} = {\text{Intercept}} + \beta {1}\left( {{\text{group}}} \right) + \beta {2}\left( {{\text{age}}\_{\text{centered}}} \right) + \beta {3}\left( {{\text{age}}\_{\text{centered}} \times {\text{group}}} \right) + {\text{e}}$$

Moreover, Pearson correlation analysis was used to investigate the relation between dystonia severity and signal intensity for each ROI and average values of 3 ROIs. All statistical analyses were performed using IBM SPSS Statistics 20 (SPSS Inc., Chicago, IL, USA) and adjusted for multiple comparison with a Benjamini–Hochberg correction. Differences were considered statistically significant at P < 0.05.

### Financial disclosure

There is no financial disclosure for all authors.

## Results

### Demographic and clinical characteristics

We recruited 18 female RTT patients with MECP2 mutations (the RTT group) and 28 age- and gender-matched healthy controls (the control group); however, one individual with RTT and two controls were excluded due to poor SWI imaging quality. The mean ages of the RTT group (n = 17) and the control group (n = 26) were 15.30 ± 8.1 years and 16.21 ± 7.9 years, respectively (p = 0.812). There was no significant difference in clinical features of speech, gross motor function, fine motor function, Rett syndrome severity, and symptoms between the two age groups in patients with RTT, although the older patients with RTT showed more impairment in each item compared with the control group (Table 1). Dystonia scales showed significantly increased dystonia severity in patients older than 10 years in both the Unified Dystonia Rating Scale (p = 0.010) and Fahn–Marsden scale (p = 0.007).

**Table 1:** Demographic and clinical features of patients with Rett Syndrome.

No	Age	Gene mutation		Speech	RSGMS				HF	RSSS level (score)	RSBQ	Fahn–Marsden scale	UDRS
					Sitting	Standing	Walking	Challenge					
1	4.2	*MECP2* c.880C>T, p.R294X		−	+	+	+	+	2	Moderate (9)	45	28	37.5
2	6.0	*MECP2* c.392 C>A		+	+	+	+	+	8	Mild (7)	30	28	42.5
3	6.2	*MECP2* c.392 C>A		+	+	+	+	+	8	Mild (3)	29	24.5	37.5
4	7.2	*MECP2* c.502 C>T p.R168X		−	+	+	+	−	3	Mild (6)	20	26.5	33.5
5	8.1	*MECP2* c.502 C>T p.R168X		−	+	+	+	−	1	Moderate (13)	38	30.5	65.5
6	8.9	*MECP2* c.808 C>T p.R270X		−	−	−	−	−	2	Severe (16)	44	53.5	54.5
7	9.1	*MECP2* c.502 C>T p.R168X		−	+	−	−	−	1	Moderate (10)	51	30.5	52.5
8	14.3	*MECP2* c.1161 C>A		−	+	−	−	−	1	Moderate (13)	34	82.5	72
9	14.2	*MECP2* c.502 C>T p.R168X		−	+	+	+	−	1	Mild (6)	31	53	66.5
10	15.6	*MECP2* c.446C>A p.S149Y		−	+	+	+	−	7	Moderate (8)	20	13	33.5
11	18.0	*MECP2* 856-859del4 AAAG del, frameshift (K286fs )		−	+	−	−	−	5	Severe (18)	56	86.5	75
12	20.8	*MECP2* frameshift insertion 38_39insTCCT (G13fs)		−	+	+	+	−	2	Moderate 10)	70	65	66
13	22.8	*MECP2* c.763C>T p.R255X		+	+	+	+	−	7	Moderate (10)	49	38.5	56
14	25.3	*MECP2* c.763C>T p.R255X		−	−	−	−	−	1	Moderate (11)	56	72.5	60.5
15	25.9	*MECP2* c. 502 C>t, p.R168X		−	+	+	+	−	2	Moderate (12)	39	59	71
16	26.9	*MECP2* mutation (losing data)		−	+	+	+	−	1	Moderate (12)	63	73	84.5
17	28.1	*MECP2* c.763C>T p.R255X		−	+	+	+	−	1	Moderate (11)	38	63	73
		Age < 10		2(29%)	6(86%)	5(71%)	5(71%)	3(43%)					
		Age > 10		1(10%)	9(90%)	7(70%)	7(70%)	0					
		Age < 10	Mean (SD)						3.57 (2.52)	9.14 (4.37)	36.71 (10.88)	31.64 (9.86)	46.21 (11.60)
		Age > 10	Mean (SD)						2.8 (2.53)	11.1 (3.17)	45.6 (15.74)	60.60 (21.82)	65.80 (13.82)
			*p* value						0.232	0.315	0.230	0.010*	0.007**

Age group < 10 years (n = 7), > 10 years (n = 10); Speech +: single word, −: mute.

*MECP2* methyl-CpG-binding protein 2, *RSGMS* Rett Syndrome Gross Motor Score, *HF* hand function scale, *RSSS* Rett Syndrome Severity Score, *RSBQ* Rett Syndrome Behaviour Questionnaire, *UDRS* Unified Dystonia Rating Scale.

*p < 0.05, **p < 0.001. Adjusted for multiple comparison with Benjamini–Hochberg correction.

### Differences in signal intensity in the deep gray matter

**Figure 1:**
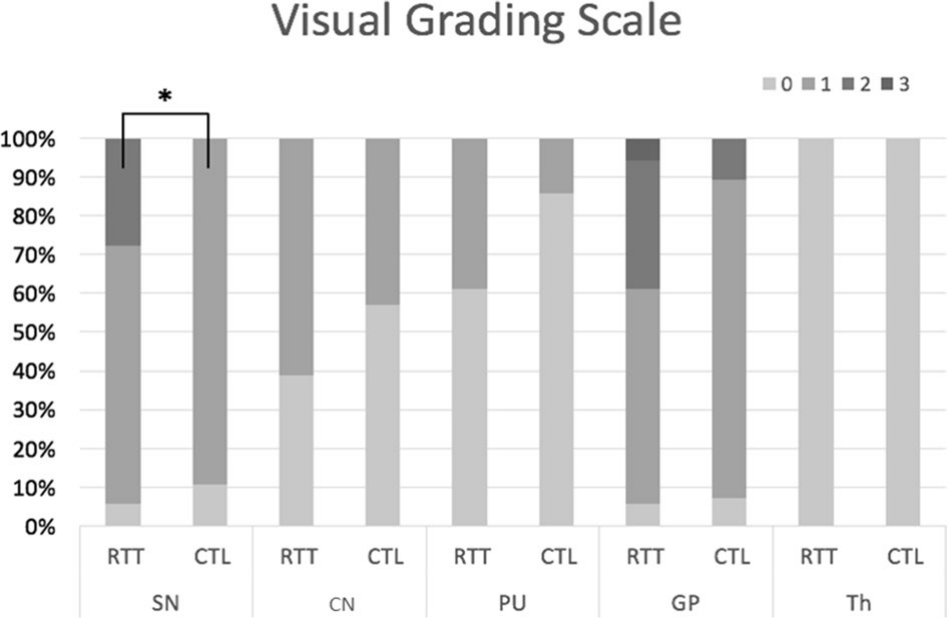
SWI hypointensity score in five deep gray matter. Stacked column chart descripted the percentage of visual grading scale for hypointensity of RTT group and control group. Significant difference was noted in substantia nigra (p = 0.014). Generally, hypointensity signal was higher in RTT group than the controls, except the signal in thalamus. (*CTL* control group, *RTT* Rett syndrome, *SN* substantia nigra, *CN* caudate nucleus, *PU* putamen, *GP* globus pallidus, *TH* thalamus).

In the present study, a visual grading scale and contrast ratio were applied to evaluate the abnormality of iron deposition in RTT. On the visual grading scale, both controls and patients with RTT had lower hypointensity scores (0 or 1) for the CN, putamen, and thalamus. Although there was no significant difference in visual grading scale between the RTT and control groups (corrected p = 0.295), patients with RTT showed a more severe hypointensity (mild: 56%; moderate: 33%; severe: 0.6%) than controls (mild: 82%; moderate: 11%) in GP. In contrast, the signal intensity in the SN differed significantly between the RTT and control groups (p = 0.014) (Fig. 1). There was no significant difference in hypointensity in the thalamus for both control and patient groups. In the contrast ratios, although mean values in the RTT group were more negative than those in the controls in all regions, only the putamen (p < 0.001), CN (p < 0.001), and average (p = 0.025) showed significantly lower values in RTT after correction (Table 2). The mean value of GP was also lower in RTT although it did not reach statistic significance (p = 0.056).

**Table 2:** Comparison of contrast ratio in five deep gray matter between patients with Rett syndrome and controls.

Deep gray matter regions	RTT		CTL		t	*p* value (2-tailed)	95% CI	
	Mean	Std. deviation	Mean	Std. deviation				
							Lower	Upper
Substantia nigra (SN)	− 0.189	0.093	− 0.165	0.094	− 0.803	0.427	− 0.082	0.036
Caudate nucleus (CN)	− 0.090	0.041	− 0.021	0.068	− 3.725	< 0.001**	− 0.106	− 0.031
Putamen	− 0.063	0.043	0.022	0.074	− 4.307	< 0.001**	− 0.125	− 0.045
Globus pallidus (GP)	− 0.213	0.121	− 0.147	0.098	− 1.963	0.056	− 0.134	0.002
Thalamus	− 0.022	0.049	0.004	0.071	− 1.330	0.191	− 0.066	0.014
Average value	− 0.154	0.079	− 0.096	0.080	− 2.332	0.025*	− 0.108	− 0.007

Average value: average values of SN, PU and GP.

*p < 0.05, **p < 0.001. Adjusted for multiple comparison with Benjamini–Hochberg correction.

The results indicated that visual grading scales may be less reliable than contrast ratios in evaluating the change in SWI.

**Figure 2:**
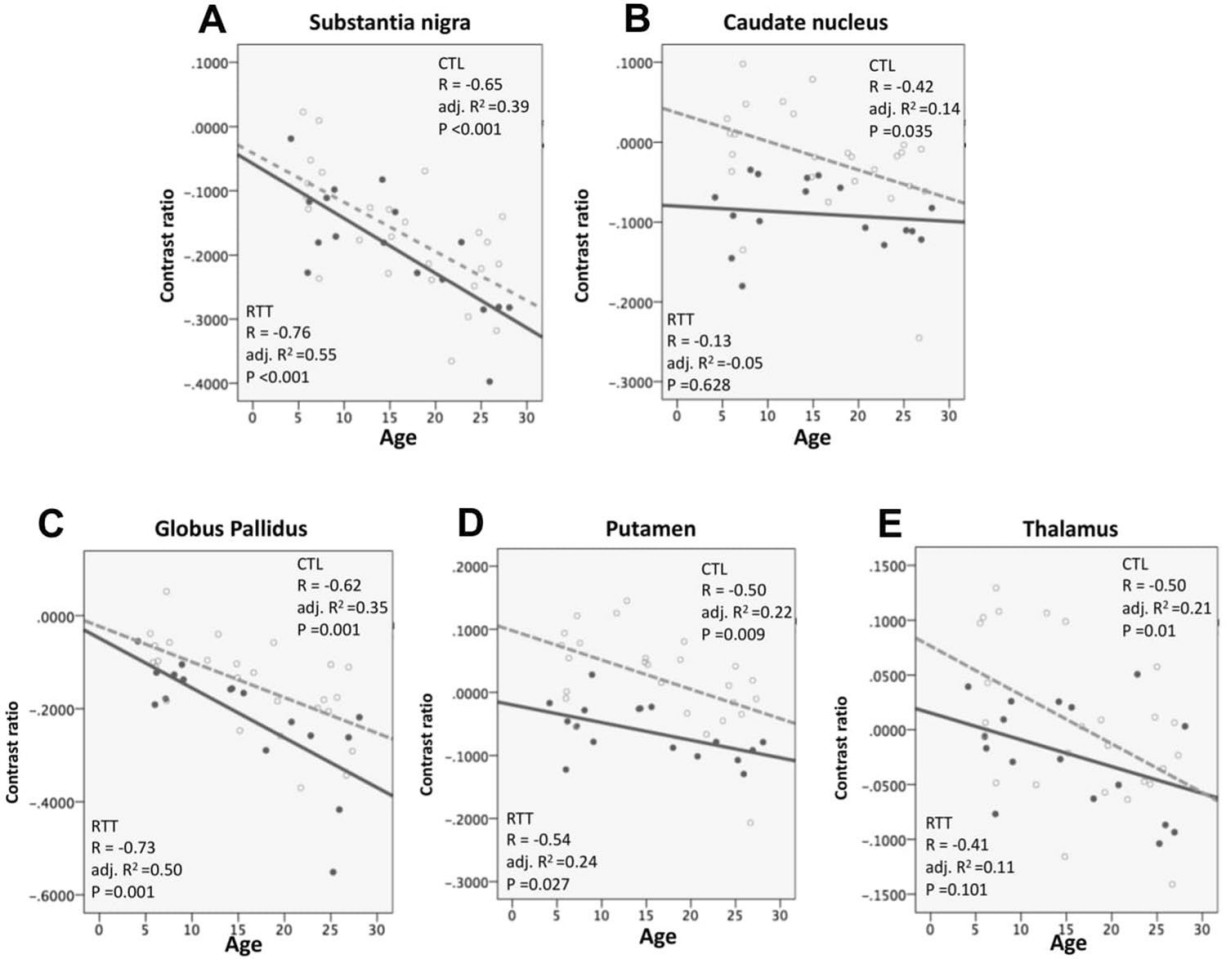
Scatter plots of the estimated age-related regression between age and contrast ratio. Regression model indicated the contrast ratio decreased significantly with age in both RTT and control groups. However, the contrast ratios were lower in RTT. The green cross/bold line was the data for RTT, and the blue square/dash line was the data for the controls.

**Figure 3:**
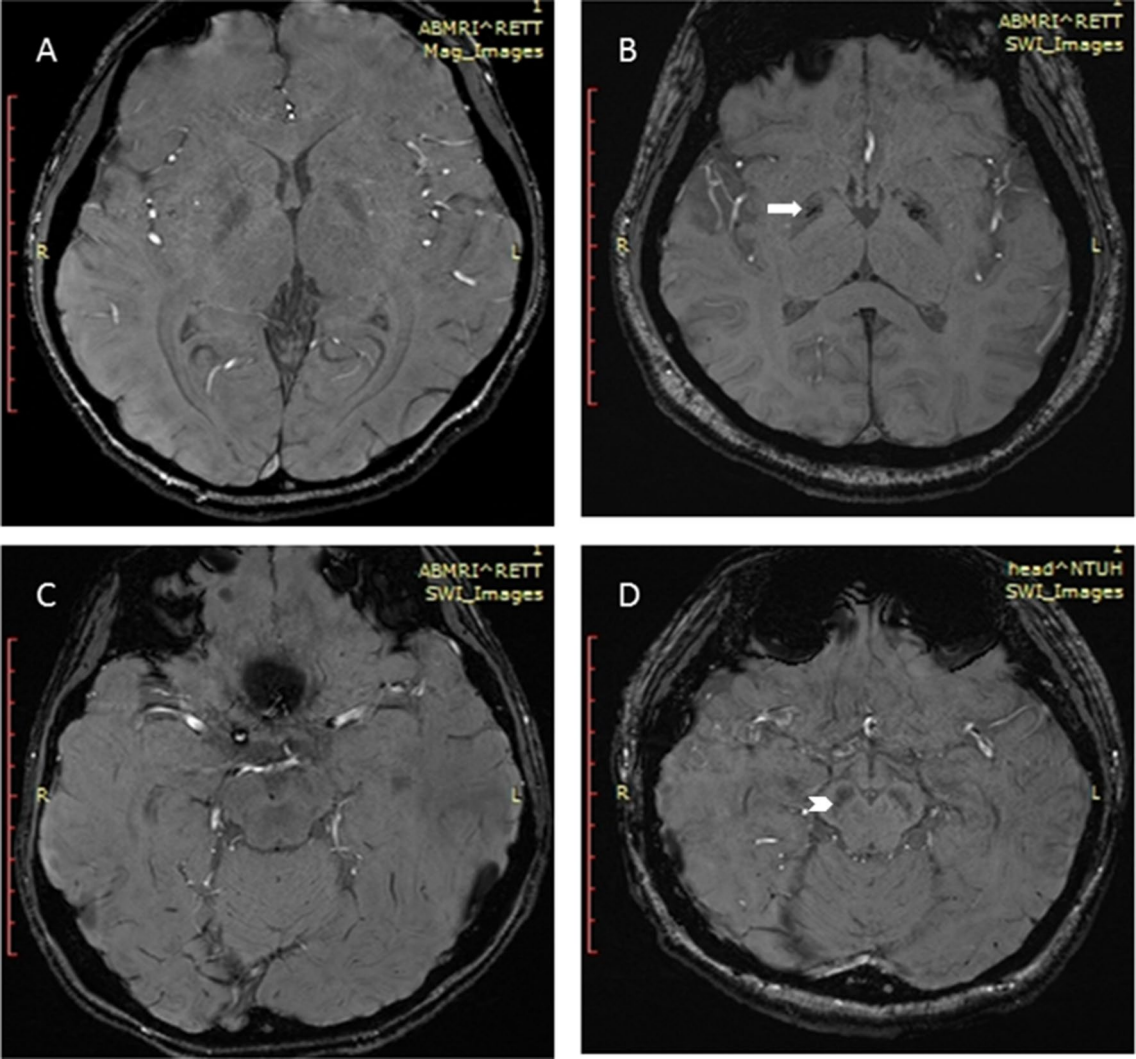
Susceptibility-weighted images in healthy control (**A**,**C**) and Rett syndrome patient (**B**,**D**) showing the relative hypointensity in globus pallidus (**B**, arrow) and substantia nigra (**D**, arrowhead) in Rett syndrome.

Age-related changes in iron accumulation were also investigated in the present study (Fig. 2). In healthy controls, age demonstrated moderate to high negative correlations with contrast ratios in all regions. In contrast, in the RTT group, age only demonstrated a moderate to high negative correlation in the SN (R = − 0.759, p < 0.001), putamen (R = − 0.536, p = 0.027), and GP (R = − 0.732, p = 0.001). In addition, the contrast ratios in individuals with RTT were significantly lower than those in healthy controls with more negative slopes in the SN and GP (Figs. 2, 3).

In a multiple regression model, age showed a significant effect on the change in iron deposition in 5 ROIs (Table 3). However, the group effect played a more important role than age in the CN and putamen (Table 3). Although the contrast ratio decreased significantly in individuals with RTT over the entire age range in GP (standardized B of group difference = − 0.328, p = 0.006), the change was more related to age factor (standardized B of age difference = − 0.547, p = 0.001) than group factor. In all multiple regression models, no interaction was found between age and group.

**Table 3:** Multiple linear regression for contrast ratio of deep gray matter with age and group.

	Standardized (B)	t	Sig	R	Adj. R^2^	F	Sig
**Substantia nigra**							
Intercept		− 11.96	< 0.001**	0.697	0.446	12.272	< 0.001**
Group	− 0.161	− 1.396	0.171				
Age	− 0.655	− 4.366	< 0.001**				
Age × group	− 0.048	− 0.318	0.752				
**Caudate nucleus**							
Intercept		− 1.824	0.076	0.600	0.31	7.301	0.001**
Group	− 0.514	− 4.004	< 0.001**				
Age	− 0.422	− 2.522	0.016*				
Age × group	0.223	1.333	0.19				
**Putamen**							
Intercept		2.177	0.036*	0.699	0.449	12.387	< 0.001**
Group	− 0.577	− 5.031	< 0.001**				
Age	− 0.49	− 3.273	0.002**				
Age × group	0.125	0.837	0.407				
**Globus pallidus**							
Intercept		− 9.032	< 0.001**	0.709	0.464	13.135	< 0.001**
Group	− 0.328	− 2.902	0.006**				
Age	− 0.547	− 3.709	0.001**				
Age × group	− 0.14	− 0.951	0.348				
**Thalamus**							
Intercept		0.495	0.624	0.509	0.202	4.54	0.008**
Group	− 0.224	− 1.624	0.112				
Age	− 0.554	− 3.074	0.004**				
Age × group	0.161	0.892	0.378				

Group was calculated as Dummy variable: control = 0, RTT = 1.

*p < 0.05, **p < 0.01.

### Correlation of clinical dystonia severity and signal intensity

Abnormal iron accumulation has been found in patients with PD and WDR45 mutations. Because adult individuals with RTT also developed more dystonia and parkinsonian features, we further investigated the correlation of iron accumulation and dystonia severity (Table 4). Both the Fahn–Marsden scale and the Unified Dystonia Rating Scale had a high correlation (R = 0.84, p < 0.001) in the present study. The Fahn-Marsden scale showed a moderately negative correlation with the contrast ratio in SN (R = − 0.518, p = 0.033), GP (R = − 0.514, p = 0.035), and the average value of 3 ROIs (R = − 0.446, p = 0.003), indicating that the contrast ratio was associated with dystonia severity. In addition, the Unified Dystonia Rating Scale showed a significant negative correlation with SN (R = − 0.529, p = 0.029). However, only the correlations between the Fahn–Marsden scales and the average values of the contrast ratios in the SN, putamen, and GP remained significant after Benjamini–Hochberg correction.

**Table 4:** The correlation between dystonia feature, age and contrast ratio of deep gray matter regions in patients with RTT.

	Age	Average	SN	CN	PU	GP	TH
**Fahn_Marsden**							
R	0.632**	− 0.446*	− 0.518*	0.137	− 0.274	− 0.514*	− 0.496
*p* value	0.006	0.003	0.033	0.599	0.288	0.035	0.053
**UDRS**							
R	0.686**	− 0.465	− 0.529*	0.198	− 0.305	− 0.403	− 0.355
*p* value	0.002	0.060	0.029	0.447	0.235	0.109	0.162
**Age**							
R	–	− 0.637**	− 0.759**	− 0.122	− .0536*	− 0.737*	− 0.404
*p* value	–	< 0.001	< 0.001	0.641	0.026	0.001	0.108

*CTL* control, *Average* average values of SN, PU and GP, *SN* substantia nigra, *CN* caudate nucleus, *PU* putamen, *GP* globus pallidus, *TH* thalamus, *UDRS* Unified Dystonia Rating Scale.

*p < 0.05, **p < 0.01. Adjusted for multiple comparison with Benjamini–Hochberg correction.

## Discussion

To our best knowledge, the present study is the first of its kind on age-related changes in SWI for RTT. We found evidence of age-related changes in iron accumulation in the deep gray matter in both RTT and control groups. Furthermore, iron deposition, revealed abnormally increased in the CN, putamen, and dopaminergic system and related grey matter (SN, putamen, and GP) in RTT, which was correlated with dystonia severity.

Abnormalities in the deep gray matter of RTT have been widely reported. A postmortem study mentioned that small cells, increased packing density, and underpigmentation in the zona compacta were shown in SN^38,39^. Neuropathological and neurochemical studies also showed abnormalities in dopaminergic terminals and deficits in fluorodopa uptake in the CN and putamen^38,40^. Moreover, structural alterations, including volume reductions, were found in the CN and thalamus^15,41^. Nevertheless, the association between these abnormalities and dystonia severity in RTT remains unclear. In our study, we found significantly increased iron deposition in the SN, putamen, and GP, and the iron deposition was correlated with dystonia severity in patients with RTT. These regions also showed more negative age-related iron changes in patients with RTT than in controls.

Dysfunctional iron regulation and abnormal iron aggregation may cause oxidative stress leading to cell death^23^.The link between oxidative stress and RTT has been extensively investigated^42–44^. One of the functions of the MECP2 gene might be protection of the CNS from hypoxia. Individuals with RTT are known to have autonomic dysfunction, characterized by dysregulated respiration and reduced arterial oxygen^45^. This might lead to hypoxic conditions and a systemic redox imbalance in the brain, leading to higher iron release^46^. Abnormal iron release may cause excessive oxidative stress, eventually resulting in extrapyramidal dysfunction, such as dystonia^47^. Along with age-related iron accumulation, dystonia may also worsen in individuals with RTT. The present study showed that individuals with RTT older than 10 years were significantly deteriorated in dystonia compared with those at a younger age, which may be related to iron accumulation in the dopaminergic system and related grey matter. This result is coincident with previous studies showing that older girls with RTT had more dystonia and gross motor deterioration^17,32^.

In healthy people, iron is distributed in a regional, age-related pattern in the extrapyramidal system. A postmortem study discovered that non-heme iron in SN and GP increased rapidly from birth to the first two decades of life; then, there was no further change in subsequent years, even though the variability of iron values in the SN increased considerably with age. On the other hand, iron accumulated more slowly in the putamen and CN of healthy people and was deposited maximally after middle age^20,48^. In the present study, individuals with RTT and healthy subjects demonstrated a similar age-related change in signal intensity in the dopaminergic system and related grey matter, but there was an increased rate of iron accumulation in individuals with RTT from a young age, indicating exacerbation of aging in these patients. The regression model showed that the group difference between RTT and controls influenced iron accumulation in the putamen and CN significantly more than age, but the alterations in these regions were not correlated with dystonia severity and other clinical presentations, possibly indicating a sign of early brain aging in RTT. Early aging had been observed in clinical characteristics of middle-age RTT. Previous studies demonstrated premature neuromuscular aging in RTT, with loss of muscle strength and muscle volume and a change in the texture of skin and hair^14,17^, compatible with the brain changes in the present study.

In our study, dystonia severity in RTT was only correlated with iron accumulation in the dopaminergic system and related grey matter (SN, putamen and GP). The damage caused by iron accumulation in the dopaminergic system and related grey matter may lead to oxidative stress, cell death, and secondary defects in axonal transport, leading to dystonia.

As this is the first investigation of iron accumulation in RTT, there were some limitations in our study. First, methodological limitations lead to differences in the results for the visual grading scale and contrast ratio. Although the visual grading scale may be influenced by the signal-to-noise ratio and lead to deviation in visual judgment, it remains the simplest and the most immediate analytical tool for clinical experts. The small sample size is the second limitation of the present study. The age range of the study also limited the interpretation of iron deposition. Another limitation of this study is that we did not have sufficient parkinsonian information on RTT. Therefore, a longitudinal follow-up study with an effective sample size and a more comprehensive clinical investigation is desired in the future.

In conclusion, the present study found abnormal iron accumulation in the deep gray matter of individuals with RTT, with exacerbated iron deposition in the putamen, CN, and dopaminergic system and related grey matter (SN, putamen, and GP). Iron accumulation in the dopaminergic system and related grey matter was also significantly correlated with dystonia severity in RTT. This result revealed a new perspective on how unusual iron accumulation accelerates neuropathological damage and aging in the RTT brain. Further longitudinal studies are mandatory for clarification of early aging and the change in iron accumulation in the brains of individuals with RTT.

## Data Availability

Anonymized data will be shared by request from any qualified investigator.
